# Same Hospital, Different Outcome: In-Hospital Mortality and Divergent Prognostic Associations in Jewish and Arab Patients with Acute Ischemic Stroke in Northern Israel

**DOI:** 10.3390/jpm16100535

**Published:** 2026-10-20

**Authors:** Chen Hanna Ryder, Yazid Badarny, Carmit Gal, Gili Barkay, Einav Levy, Rima Nassar, Samih Badarny, Radi Shahien

**Affiliations:** 1 The Brain and Behavior Research Institute, Western Galilee College, Akko 2412101, Israel; 2 Department of Neurosurgery, Rambam Medical Center, Haifa 3109601, Israel; y_badarny@rambam.health.gov.il (Y.B.);; 3 Department of Psychology, Kinneret College on the Sea of Galilee, Jordan Valley 1513200, Israel; 4 Research Center for Innovation in Social Work, Department of Social Work, Tel-Hai University of Kiryat Shmona in the Galilee, Qiryat Shemona 1220800, Israel; leviein@telhai.ac.il; 5 Department of Surgery, Bnai Zion Medical Center, Haifa 3339473, Israel; rima.nassar@b-zion.org.il; 6 Neurology Department, Galilee Medical Center, Nahariya 2210001, Israel; 7 Azrieli Faculty of Medicine, Bar-Ilan University, Safed 1311502, Israel; radis@ziv.gov.il; 8 Research Authority, Ziv Medical Center, Safed 1311001, Israel

**Keywords:** acute ischemic stroke, in-hospital mortality, personalized medicine, risk stratification, C-reactive protein, prognostic heterogeneity, model transportability, population groups, Israel, Firth penalized regression

## Abstract

**Background/Objectives:** Individualized risk estimation after stroke depends on prognostic models, which may not transfer between populations. Jewish and Arab patients with acute stroke in the Western Galilee share one regional center and its protocols. We examined whether mortality, prognostic associations of admission variables and pooled-model calibration differ between them. **Methods:** This retrospective single-center cohort study included adults with non-lacunar acute ischemic stroke and moderate or greater vessel occlusion admitted to the Galilee Medical Center, Israel, from March 2023 to February 2025. The outcome was in-hospital death. Mortality was compared before and after adjustment for age, sex and stroke severity, and prognostic heterogeneity was tested with interaction terms in Firth penalized regression. **Results:** Of 499 patients, 289 were Jewish and 210 Arab; Jewish patients were older (mean 72.1 versus 67.5 years) and less often current smokers, with similar sex distribution and stroke severity. Mortality was 11.1% in Jewish and 4.8% in Arab patients (risk ratio 2.33, 95% confidence interval (CI) 1.17 to 4.62; adjusted odds ratio 2.05, 95% CI 0.97 to 4.34). In a joint test of age, sex and C-reactive protein (CRP), prognostic associations differed between groups (*p* = 0.012), mainly because CRP was more weakly associated with death in Jewish patients; white blood cell count showed a similar exploratory pattern. A pooled model without a group term tended to underpredict deaths in Jewish and overpredict them in Arab patients. **Conclusions:** Within one stroke service, mortality and the prognostic weight of admission variables differed between population groups. These exploratory findings require replication and, if confirmed, would support population-aware validation and recalibration of prognostic models. They concern risk estimation, not treatment, which should follow the same criteria in both groups.

## 1. Introduction

Personalized medicine in acute stroke rests on the assumption that a patient’s measured characteristics can be translated into an individual estimate of risk, and that this estimate is accurate enough to inform decisions about monitoring intensity, escalation of care and goals-of-care discussions. That assumption is only as good as the prognostic model behind it. A model estimated in one population encodes both the case mix of that population and the strength of each predictor-outcome relationship within it, and neither necessarily carries over to a different setting [1,2]. Reproducibility is performance in a new sample from the same population, whereas transportability is performance in a related but different population [1]. Distinguishing the two is therefore a practical prerequisite for personalized risk stratification rather than a statistical afterthought.

Stroke offers an unusually clear test of this problem. Stroke is the second leading cause of death worldwide, ischemic stroke accounts for the majority of cases, and the absolute burden continues to rise despite falling age-standardized rates [3]. Reperfusion therapy has transformed early management [4], yet outcome after treatment remains heterogeneous. Several bedside scores estimate early death or severe disability after ischemic stroke from variables available at admission, among them iScore, the Acute Stroke Registry and Analysis of Lausanne (ASTRAL) score and the PLAN score (preadmission comorbidities, level of consciousness, age and neurologic deficit) [5,6,7]. Risk scores are used across populations that differ substantially in age structure, vascular risk-factor profile and baseline inflammatory state, and whether a single score serves all of them is rarely examined directly.

C-reactive protein (CRP) illustrates the difficulty. CRP measured after acute ischemic stroke is associated with poor functional outcome and death [8,9], including among patients with lacunar infarction [10], and baseline CRP is associated with incident vascular events and vascular mortality in a large individual-participant meta-analysis [11]. However, adding inflammatory markers to clinical prediction models has produced little improvement in discrimination, calibration or reclassification [12], and baseline CRP concentrations differ substantially between population groups [13,14,15]. A marker whose distribution and whose association with outcome both vary between populations is precisely the kind of variable for which transportability cannot be assumed.

Northern Israel provides a setting in which this question can be asked with unusually few competing explanations. Jewish and Arab residents of the Western Galilee live in the same region, in largely separate localities, and are covered by the same statutory health insurance [16]. For acute stroke they are admitted to a single regional center that applies one set of protocols. The two groups nevertheless differ systematically in age at stroke onset and in vascular risk-factor burden: Arab patients present younger and with more diabetes mellitus and smoking, in national registry data as well as in single-center series [17,18,19,20]. Similar patterns have been reported for intracranial hemorrhage in the same region [20], and clinically meaningful heterogeneity exists even within the Arab population group, between Druze, Muslim, Bedouin and Christian subgroups [21]. An analysis of acute stroke treatment among revascularized patients in the Israeli national registry found comparable rates of interventional treatment, comparable favorable outcome and comparable mortality between the groups, although intravenous thrombolysis was given less often to Arab patients [18]. Against that background, any residual difference in in-hospital death is worth quantifying carefully.

Throughout this paper, population group refers to the Jewish or Arab designation recorded in administrative and clinical records, and we use this term consistently in preference to ethnicity or ethnic group. It is a social and administrative category that captures a complex bundle of environment, exposure, culture, language, socioeconomic position and health-service interaction. It is not a genetic ancestry variable, and we do not treat it as a proxy for one [22,23,24]. The question we ask is not whether the two groups are biologically different, but whether a prognostic model estimated on the combined cohort would serve both of them equally well.

We therefore had three aims. First, to quantify the difference in in-hospital mortality after acute ischemic stroke between Jewish and Arab patients treated at one regional center, before and after adjustment for case mix. Second, to test formally, using explicit interaction terms rather than group-specific significance, whether the prognostic associations of age, sex and admission CRP differ between the two groups. Third, to examine how a single pooled prognostic model calibrates within each group, as a direct measure of what population-blind risk estimation would cost in this setting. Together these questions ask whether individualized risk estimation, which is the operational core of personalized medicine, holds its accuracy across the population groups that a single service actually treats.

## 2. Materials and Methods

### 2.1. Study Design and Setting

We conducted a retrospective observational cohort study at the Galilee Medical Center, Nahariya, a government tertiary hospital that serves as the regional stroke center for the Western Galilee. The catchment area includes both Jewish and Arab localities, and both population groups are treated by one stroke service under a single set of acute care protocols, including intravenous thrombolysis and endovascular therapy.

All patients entered in the departmental stroke database between March 2023 and February 2025 were eligible. The database records adults admitted to the stroke service with imaging-confirmed ischemic stroke and was compiled with the clinical focus described in Section 2.2, so the cohort represents non-lacunar stroke with moderate or greater vessel occlusion rather than the full spectrum of acute ischemic stroke. The study was conducted in accordance with the Declaration of Helsinki and was approved by the Institutional Review Board of the Galilee Medical Center (protocol code 0008-23-NHR, approved 26 March 2023), which granted a waiver of informed consent for the retrospective analysis of de-identified routinely collected data. Reporting follows the Strengthening the Reporting of Observational Studies in Epidemiology (STROBE) recommendations for cohort studies [25], and the prediction-model components additionally follow the Transparent Reporting of a multivariable prediction model for Individual Prognosis Or Diagnosis (TRIPOD) recommendations [26].

The stroke database from which this cohort is drawn was built by our group to support several distinct analyses, each addressing different variables and a different research question. One of these, comparing anterior with posterior circulation stroke, has been published [27]; it examined a different set of variables and did not analyze mortality or population group. No result reported in the present paper has been published previously.

### 2.2. Study Population

Eligible patients were aged 18 years or older, had acute ischemic stroke confirmed by clinical assessment together with non-contrast computed tomography and/or magnetic resonance imaging, and were admitted directly to the stroke service. All patients also underwent computed tomography angiography (CTA). The diagnosis and classification of each patient were made by senior neurologists of the Neurology Department on the basis of this clinical and imaging assessment. The database from which this cohort is drawn was compiled to characterize territorial ischemic stroke, and three categories were therefore excluded when it was assembled: transient ischemic attack without imaging-confirmed infarction, lacunar infarction, and ischemic stroke with only mild vessel occlusion, so that only strokes with moderate or greater vessel occlusion were entered [27]. Patients with primary intracerebral or subarachnoid hemorrhage and clinically diagnosed stroke mimics were likewise not included. Vessel occlusion was graded on CTA according to the degree of luminal narrowing (stenosis) of the artery supplying the infarcted territory: narrowing of 50% to 70% was considered moderate and narrowing above 70% severe, so that moderate or greater occlusion denotes narrowing of at least 50%. Because the neurologists’ classification determined entry into the database, it is not held as a separate variable in the analysis dataset. Stroke etiology was not an eligibility criterion, so cardioembolic strokes were included when they met the other criteria. The cohort is consequently a defined clinical subgroup and not a consecutive series of all acute ischemic strokes; results apply to non-lacunar stroke with moderate or greater vessel occlusion managed at a thrombectomy-capable regional center. Patients whose recorded population group was neither Jewish nor Arab were not eligible for the present comparison.

### 2.3. Variables and Definitions

Population group was taken from the designation recorded in the hospital’s administrative record, which follows the national population registry. No genetic, ancestry or subgroup information was available, and Arab subgroups (Muslim, Christian, Druze and Bedouin) could not be distinguished in this dataset.

Stroke severity was scored with the National Institutes of Health Stroke Scale (NIHSS) by trained clinical staff at admission and again at the last in-hospital assessment, which was the discharge assessment in patients discharged alive. Vascular risk factors (hypertension, diabetes mellitus, hyperlipidemia and current smoking) were recorded as present or absent according to documented diagnosis or treatment at the time of admission. A composite variable indicating three or more of these four risk factors was derived for descriptive purposes only and was not entered into any regression model.

C-reactive protein (CRP, mg/L) and white blood cell count (WBC, ×10^9^/L) were measured in the hospital’s central laboratory on venous blood drawn as part of the routine admission work-up. Prothrombin time, activated partial thromboplastin time and the international normalized ratio (INR) were recorded from the same sample. Acute reperfusion treatment was recorded as intravenous thrombolysis and as mechanical thrombectomy, each as a binary variable. Infarct territory was classified as anterior or posterior circulation, and hemispheric side was recorded as left, right or bilateral/undetermined.

### 2.4. Outcomes

The primary outcome was in-hospital all-cause death, ascertained from the discharge record. Length of stay and discharge destination (home, institutional care or death) were recorded as descriptive secondary outcomes. Post-discharge follow-up was not available and no post-discharge outcome is reported.

### 2.5. Statistical Analysis

Analyses were performed in Python 3.11.14 using pandas 2.3.3, NumPy 2.3.4, SciPy 1.16.3, statsmodels 0.14.5 and Matplotlib 3.10.7. Additional analyses were performed in Python 3.11.15 using pandas 3.0.2, NumPy 2.4.4, SciPy 1.17.1, statsmodels 0.15.0, scikit-learn 1.8.0 and Matplotlib 3.10.9, in which the principal estimates of the original analysis were reproduced exactly. Firth penalized logistic regression and penalized profile-likelihood confidence intervals (CIs) were implemented directly and verified against an independent data-augmentation algorithm for the same estimator, which reproduced every coefficient to within 1 × 10^−10^.

Continuous variables are summarized as mean with standard deviation and compared with independent-samples *t* tests, or as median with interquartile range (IQR) and compared with Mann-Whitney *U* tests where the distribution was markedly skewed. Categorical variables are summarized as counts with percentages and compared with Pearson’s chi-square test, or with Fisher’s exact test where any expected count was below five. Binomial proportions are reported with Wilson confidence intervals, and matched pairs were compared with the exact McNemar test. Effect sizes are Cohen’s *d* for variables compared with *t* tests and the phi coefficient or Cramér’s V for categorical variables; no effect size is reported for rank-based comparisons. To examine whether the baseline differences between the groups (Table 1) were independent of one another, the baseline characteristics and acute treatments were also entered together into a multivariable logistic regression model with population group (Jewish versus Arab) as the dependent variable (Table 2). Two variables were not entered: hemispheric side, because 64 of the 65 posterior-circulation strokes were coded as bilateral or undetermined, so that this category could not be separated from infarct territory; and prothrombin time, because the international normalized ratio, which was entered, is derived from it. Crude risk ratios and risk differences with 95% confidence intervals were computed directly from the two-by-two mortality table. Mortality was also described within three strata of admission CRP (at or below 3 mg/L, above 3 up to 50 mg/L, and above 50 mg/L), with Wilson confidence intervals for each proportion and Fisher’s exact test comparing the groups within each stratum; these three comparisons are descriptive and are not adjusted for multiple testing.

Because the distribution of CRP was strongly right-skewed, CRP was analyzed on the base-10 logarithmic scale (log_10_ CRP), so that each odds ratio (OR) for CRP refers to a 10-fold increase in concentration, for example from 5 to 50 mg/L; the distribution of CRP concentrations and their clinical interpretation are reviewed elsewhere [28]. Seven patients had a recorded CRP of 0 mg/L and are therefore excluded from analyses using log_10_ CRP; a sensitivity analysis using log_2_(CRP + 1), which retains these patients and is scaled per doubling of (CRP + 1), is reported. White blood cell count is summarized on the base-10 logarithmic scale in Table 1 and was entered in regression models per 1 × 10^9^/L, the international normalized ratio per 0.1, and prothrombin and activated partial thromboplastin times per second.

The primary multivariable model contained age, sex, population group and admission NIHSS. Age, sex and stroke severity were chosen because they are established admission predictors of death and poor outcome after ischemic stroke: age and stroke severity are components of the iScore, ASTRAL and PLAN scores, and sex is a component of the iScore [5,6,7]. Population group was the exposure of interest. The model was specified before the multivariable analyses were run. With 42 deaths, it was limited to these four predictors to reduce the risk of overfitting rather than to satisfy any fixed events-per-variable rule, since the adequacy of a given sample depends on the number of predictors, the outcome proportion and the expected predictive performance rather than on a universal ratio [29,30,31]. Optimism was assessed by bootstrap resampling. The other admission variables were examined in three further ways. The unadjusted association of every recorded admission variable with in-hospital death is reported. An extended model added diabetes mellitus and current smoking, which are more common among Arab patients in national data [17,18], and log_10_ CRP, the focus of this study. An exploratory specification-robustness model contained eleven predictors: age, sex, population group, hypertension, diabetes mellitus, hyperlipidemia, current smoking, admission NIHSS, log_10_ CRP, intravenous thrombolysis and mechanical thrombectomy. All coefficients of this third model are reported, but with fewer than four deaths per predictor they are interpreted with caution, and the model serves mainly as a check on the stability of the population-group estimate. In all models, female sex is presented as the index category and male sex as the reference. Model fit is reported as Nagelkerke R^2^ and, for completeness, the Hosmer-Lemeshow test, which is retained only as a coarse check for gross lack of fit; the calibration curves, observed-to-expected ratios and calibration intercepts and slopes reported below are the informative measures of calibration [32].

Prognostic heterogeneity was assessed by explicit interaction terms in the pooled cohort, not by comparing whether a coefficient reached significance in one group and not in the other, which is not a valid test of difference [33]. Three product terms, between population group and age, sex and log_10_ CRP, were defined for this analysis. The three variables were chosen because, for each of them, published evidence gave a specific reason to expect that its association with in-hospital death might differ between the two groups. This is the situation in which a prognostic association estimated in one population cannot be assumed to hold in another [1]. For age, the two groups differ systematically in age at stroke onset, Arab patients presenting younger in national stroke registry and survey data [18,19], so that the association of age with death is estimated over different age ranges in the two groups. For sex, an earlier study of ischemic stroke among Arab patients in northern Israel found marked sex differences in stroke severity and stroke subtype among Druze patients [21], which gave reason to test whether sex carries the same prognostic information in Arab as in Jewish patients. For CRP, baseline concentrations differ between population groups [13,14,15], and CRP measured after acute ischemic stroke is associated with death [8,9], so its prognostic weight could not be assumed to be the same in both groups. The number of product terms was limited to these three to preserve power: with 41 deaths, 10 of them among Arab patients, each additional term would have diluted the joint test and increased the risk of a chance finding. Every interaction model was also adjusted for admission NIHSS.

The three terms were tested jointly and individually as ratios of odds ratios. Because the sex term approaches separation, each interaction model was fitted twice: by Firth penalized likelihood, with profile-likelihood confidence intervals and penalized likelihood-ratio *p* values, which provides the primary estimates, and by maximum likelihood, with Wald confidence intervals and Wald *p* values, which are given alongside them. In the sensitivity analyses, where several strata approach separation, penalized estimates are reported, except under multiple imputation, for which maximum-likelihood estimates were pooled. The remaining admission variables were then examined in an exploratory screen, in which the interaction between population group and each variable was tested in turn in a separate penalized model adjusted for age, sex and admission NIHSS. Hemispheric side, a three-category variable, was tested with a penalized likelihood-ratio test on two degrees of freedom (df). These 13 tests are hypothesis-generating, and Benjamini-Hochberg false-discovery-rate values across them are reported alongside their *p* values (Table S1). For a variable with evidence of an interaction in the screen, robustness was examined post hoc in five ways: after adjustment for log_10_ CRP; in a joint model that added its product term to a model containing age, sex, admission NIHSS, log_10_ CRP, the variable itself, population group and the CRP product term; on a logarithmic scale; after winsorization at the 95th percentile; and after exclusion of extreme values.

Group-stratified models are reported for interpretation. Because only 10 deaths occurred among Arab patients, unpenalized stratified estimation is exposed to sparse-data bias and to inflated effect estimates [34]. The adjusted stratified models were therefore restricted to the four variables of the interaction model (age, sex, admission NIHSS and log_10_ CRP), so that the group-specific estimates can be read alongside the interaction estimates, and were estimated by Firth penalized likelihood [35,36,37]. For completeness, the unadjusted penalized association of every other recorded admission variable with in-hospital death is also reported within each group. Because Firth estimation biases predicted probabilities toward one half under rare outcomes [37], penalized models are used for effect estimation only and not for absolute risk prediction.

To quantify the practical cost of population-blind risk estimation, a pooled model containing the same four admission variables (age, sex, admission NIHSS and log_10_ CRP), and no population-group term, was fitted to the 487 patients with CRP above 0 mg/L, so that the calibration analysis concerns the model whose coefficients were compared between the groups. Its linear predictor was then evaluated separately within each group by the ratio of observed to expected deaths and by the calibration intercept and calibration slope obtained from a logistic recalibration model regressing the outcome on the linear predictor [32,38]. Because the model was both fitted and evaluated in this cohort, this is an internal, exploratory comparison of subgroup calibration and not an external validation; it quantifies how a pooled model behaves in each group here, not how it would transport to another setting. The calibration intercepts were compared by adding a population-group term to that recalibration model, and the calibration slopes by adding a product term between the linear predictor and population group; both were tested with likelihood-ratio tests. Discrimination is reported as the C-statistic with a bootstrap percentile confidence interval, and optimism was estimated by bootstrap resampling [39,40].

The sensitivity analyses were: restriction of CRP to values at or below 100 mg/L and at or below 50 mg/L; winsorization of CRP at the 95th percentile; the log_2_(CRP + 1) transformation; a binary CRP threshold of 3 mg/L; exclusion of patients who received intravenous thrombolysis; exclusion of patients who received mechanical thrombectomy; restriction to patients who received neither thrombolysis nor thrombectomy; restriction to patients aged 50 years or older; 1:1 nearest-neighbor propensity-score matching without replacement [41,42]; and multiple imputation by chained equations (20 imputations, Rubin’s rules) for models containing CRP [43]. In the matching, each Arab patient was matched to the Jewish patient with the closest logit propensity score within a caliper of 0.2 standard deviations of the logit propensity score, on age, sex, hypertension, diabetes mellitus, hyperlipidemia, current smoking, admission NIHSS and log_10_ CRP, and balance was assessed by standardized mean differences. The matched comparison was also summarized as the conditional odds ratio, the ratio of the two types of discordant pairs, with an exact confidence interval. Tests were two-sided with alpha of 0.05, and exact *p* values are reported to three decimal places, with values below 0.001 given as *p* < 0.001. Multiplicity in the interaction analysis was addressed by design: the three product terms were fixed in advance of the interaction analysis and tested jointly with a single likelihood-ratio test, and the individual ratios of odds ratios are interpreted as components of that test rather than as independent hypotheses.

## 3. Results

### 3.1. Cohort

The extracted database contained 500 records, of which one was empty and was excluded. The analytic cohort therefore comprised 499 patients: 289 Jewish patients (57.9%) and 210 Arab patients (42.1%). The departmental database from which the records were drawn contains only imaging-confirmed ischemic strokes, and no record carried a population group other than Jewish or Arab, so none of the exclusion criteria in Section 2.2 removed a patient at this stage. No patient was lost to in-hospital follow-up, because the outcome was ascertained from the same admission record. Forty-two patients (8.4%) died during the index admission.

### 3.2. Baseline Characteristics

Baseline characteristics are shown in Table 1. Jewish patients were older at presentation than Arab patients (72.1 ± 11.6 versus 67.5 ± 13.8 years; *p* < 0.001; Cohen’s *d* = 0.36), and more were older than 70 years (59.9% versus 43.8%; *p* < 0.001). The proportion of women did not differ (47.4% versus 45.7%; *p* = 0.709). Current smoking was half as common among Jewish patients (17.6% versus 34.8%; *p* < 0.001), and fewer Jewish patients carried three or more of the four recorded vascular risk factors (31.6% versus 43.5%; *p* = 0.006). Diabetes mellitus was less frequent among Jewish patients, although the difference did not reach conventional significance (34.9% versus 43.3%; *p* = 0.057). Hypertension and hyperlipidemia were similarly distributed.

Admission stroke severity did not differ between the groups (NIHSS 13.0 ± 5.4 versus 12.5 ± 5.3; *p* = 0.325), nor did infarct territory or hemispheric side. Among the 457 patients discharged alive, the last recorded NIHSS was similar (5.20 ± 4.69 versus 6.05 ± 5.36; *p* = 0.073), while the improvement from admission was slightly greater in Jewish patients (7.52 ± 5.45 versus 6.41 ± 5.80; *p* = 0.037); because this comparison is conditioned on survival and the two groups had different mortality, we do not interpret it further. Rates of acute reperfusion treatment were similar for intravenous thrombolysis (32.5% versus 36.7%; *p* = 0.336) and for mechanical thrombectomy (27.4% versus 29.2%; *p* = 0.667).

Admission CRP was lower in Jewish patients on both the raw and the logarithmic scale (median 4.8 mg/L, IQR 2.0 to 9.7, versus 5.5 mg/L, IQR 2.7 to 13.2; *p* = 0.039; log_10_ CRP 0.70 ± 0.52 versus 0.83 ± 0.54; *p* = 0.007), corresponding to geometric means of 5.0 and 6.8 mg/L. White blood cell count, prothrombin time, activated partial thromboplastin time and the international normalized ratio did not differ between the groups.

When the baseline characteristics and acute treatments were entered together into a multivariable model of population group (Table 2), three differences remained independent of the others. Jewish patients were older (adjusted OR 1.023 per year, 95% CI 1.005 to 1.041; *p* = 0.013), were less often current smokers (0.43, 95% CI 0.26 to 0.71; *p* = 0.001) and had lower admission CRP (0.51 per 10-fold increase, 95% CI 0.34 to 0.74; *p* < 0.001). The lower prevalence of diabetes mellitus among Jewish patients did not reach significance after adjustment (0.68, 95% CI 0.45 to 1.04; *p* = 0.076), and none of the other characteristics differed.

**Table 1:** Baseline characteristics, treatment and outcomes by population group.

Variable	Total (*n* = 499)	Jewish (*n* = 289)	Arab (*n* = 210)	*p*	Effect Size
**Demographics**					
Age, years, mean ± SD	70.16 ± 12.74	72.09 ± 11.55	67.51 ± 13.81	<0.001	0.36
Age > 70 years, *n* (%)	265 (53.1)	173 (59.9)	92 (43.8)	<0.001	0.159
Female sex, *n* (%)	233 (46.7)	137 (47.4)	96 (45.7)	0.709	0.017
**Vascular risk factors**					
Hypertension, *n* (%) ^f^	369 (74.1)	216 (74.7)	153 (73.2)	0.700	0.017
Diabetes mellitus, *n* (%)	192 (38.5)	101 (34.9)	91 (43.3)	0.057	0.085
Hyperlipidemia, *n* (%) ^f^	315 (63.3)	180 (62.5)	135 (64.3)	0.683	0.018
Current smoking, *n* (%)	124 (24.8)	51 (17.6)	73 (34.8)	<0.001	0.196
≥3 vascular risk factors, *n* (%) ^a,f^	182 (36.6)	91 (31.6)	91 (43.5)	0.006	0.122
**Stroke characteristics**					
NIHSS at admission, mean ± SD	12.80 ± 5.33	13.00 ± 5.37	12.52 ± 5.26	0.325	0.09
NIHSS at admission, median (IQR)	14.0 (8.0–17.0)	14.0 (9.0–17.0)	13.0 (8.0–17.0)	0.344	—
NIHSS at last assessment, mean ± SD ^b^	5.57 ± 5.01	5.20 ± 4.69	6.05 ± 5.36	0.073	0.17
Improvement in NIHSS, mean ± SD ^b^	7.03 ± 5.63	7.52 ± 5.45	6.41 ± 5.80	0.037	0.20
Anterior circulation, *n* (%)	434 (87.0)	253 (87.5)	181 (86.2)	0.658	0.020
Hemispheric side, *n* (%) ^e^				0.418	0.059
Left	231 (46.3)	141 (48.8)	90 (42.9)		
Right	202 (40.5)	111 (38.4)	91 (43.3)		
Bilateral or undetermined	66 (13.2)	37 (12.8)	29 (13.8)		
**Laboratory values on admission**					
CRP, mg/L, median (IQR) ^c^	5.0 (2.4–11.0)	4.8 (2.0–9.7)	5.5 (2.7–13.2)	0.039	—
log_10_ CRP, mean ± SD ^d^	0.76 ± 0.53	0.70 ± 0.52	0.83 ± 0.54	0.007	0.25
CRP, mg/L, geometric mean ^d^	5.7	5.0	6.8	—	—
WBC, ×10^9^/L, median (IQR)	9.0 (7.0–11.0)	9.0 (7.0–11.0)	9.0 (7.0–11.0)	0.726	—
log_10_ WBC, mean ± SD	0.95 ± 0.15	0.95 ± 0.15	0.95 ± 0.16	0.888	0.01
INR, mean ± SD ^f^	1.06 ± 0.14	1.06 ± 0.14	1.05 ± 0.15	0.499	0.06
PT, s, mean ± SD ^f^	12.67 ± 4.90	12.61 ± 3.13	12.76 ± 6.61	0.745	0.03
aPTT, s, mean ± SD ^f^	30.53 ± 7.39	30.16 ± 6.58	31.03 ± 8.38	0.195	0.12
**Acute treatment**					
Intravenous thrombolysis, *n* (%)	171 (34.3)	94 (32.5)	77 (36.7)	0.336	0.043
Mechanical thrombectomy, *n* (%) ^f^	140 (28.2)	79 (27.4)	61 (29.2)	0.667	0.019
**Outcomes**					
Length of stay, days, median (IQR) ^f^	9.0 (6.0–17.0)	10.0 (6.0–17.0)	9.0 (5.0–15.0)	0.146	—
Discharge destination, *n* (%) ^e^				0.022	0.123
Home	233 (46.7)	125 (43.3)	108 (51.4)		
Institutional care	224 (44.9)	132 (45.7)	92 (43.8)		
Died in hospital	42 (8.4)	32 (11.1)	10 (4.8)		
In-hospital death, *n* (%)	42 (8.4)	32 (11.1)	10 (4.8)	0.012	0.112

aPTT, activated partial thromboplastin time; CRP, C-reactive protein; INR, international normalized ratio; IQR, interquartile range; NIHSS, National Institutes of Health Stroke Scale; PT, prothrombin time; SD, standard deviation; WBC, white blood cell count. Continuous variables were compared with independent-samples *t* tests and are reported with the absolute value of Cohen’s *d*; variables reported as median (IQR) were compared with the Mann-Whitney *U* test. Categorical variables were compared with Pearson’s chi-square test and are reported with the phi coefficient, or with Cramér’s V where marked ^e^; Cohen’s *d* is given to two decimal places and phi or Cramér’s V to three. Denominators are 499, 289 and 210 except where a footnote states otherwise. The adjusted comparison of these characteristics between the groups is shown in Table 2. ^a^ Three or more of hypertension, diabetes mellitus, hyperlipidemia and current smoking; available for 497 patients. ^b^ Restricted to the 457 patients discharged alive (257 Jewish, 200 Arab), because a repeat NIHSS in patients who died in hospital reflects the last assessment before death rather than a discharge state; improvement is admission NIHSS minus last-assessment NIHSS in the same patients. ^c^ Available for 494 patients (287 Jewish, 207 Arab). ^d^ Computed among the 487 patients with CRP above 0 mg/L; the geometric-mean row is the back-transform of the log_10_ CRP row above it and shares its *p* value. ^e^ *p* value and effect size refer to the three-category distribution as a whole. ^f^ Denominators after missing values: hypertension 289 Jewish and 209 Arab; hyperlipidemia 288 and 210; ≥3 vascular risk factors 288 and 209; mechanical thrombectomy 288 and 209; length of stay 289 and 209; INR 285 and 204; prothrombin time 288 and 209; activated partial thromboplastin time 289 and 209. Exact *p* values are given to three decimal places; *p* values below 0.001 are shown as <0.001.

**Table 2:** Multivariable logistic regression of population group on baseline characteristics and acute treatment.

Variable	Adjusted OR for Jewish Versus Arab Population Group (95% CI)	*p*
Age, per year	1.023 (1.005–1.041)	0.013
Female sex	0.81 (0.53–1.23)	0.323
Hypertension	0.98 (0.59–1.62)	0.933
Diabetes mellitus	0.68 (0.45–1.04)	0.076
Hyperlipidemia	1.01 (0.65–1.58)	0.964
Current smoking	0.43 (0.26–0.71)	0.001
NIHSS at admission, per point	0.991 (0.952–1.032)	0.672
Anterior circulation	1.23 (0.67–2.29)	0.505
log_10_ CRP, per 10-fold increase	0.51 (0.34–0.74)	<0.001
WBC, per 1 × 10^9^/L	1.041 (0.980–1.105)	0.190
INR, per 0.1	1.068 (0.925–1.233)	0.371
aPTT, per s	0.982 (0.956–1.008)	0.173
Intravenous thrombolysis	0.71 (0.46–1.08)	0.108
Mechanical thrombectomy	1.09 (0.68–1.74)	0.718
Patients (Jewish/Arab)	473 (277/196)	—
C-statistic	0.679	—

aPTT, activated partial thromboplastin time; CI, confidence interval; CRP, C-reactive protein; INR, international normalized ratio; NIHSS, National Institutes of Health Stroke Scale; OR, odds ratio; WBC, white blood cell count. Values are odds ratios of belonging to the Jewish rather than the Arab population group, from a single multivariable logistic regression model containing all the listed characteristics and fitted to the 473 patients with complete data (277 Jewish, 196 Arab). An odds ratio above 1 indicates a characteristic that was more common, or a value that was higher, among Jewish patients after adjustment for the others, and an odds ratio below 1 the reverse. Male sex is the reference category for female sex. log_10_ CRP is scaled per 10-fold increase in CRP. Hemispheric side was not entered because 64 of the 65 posterior-circulation strokes were coded as bilateral or undetermined, and prothrombin time was not entered because the INR is derived from it. Likelihood-ratio test of the model χ^2^ = 44.59, df = 14, *p* < 0.001. Exact *p* values are given to three decimal places; *p* values below 0.001 are shown as <0.001.

### 3.3. In-Hospital Mortality

In-hospital mortality was 11.1% (32/289; 95% CI 8.0% to 15.2%) among Jewish patients and 4.8% (10/210; 95% CI 2.6% to 8.5%) among Arab patients. The risk ratio for Jewish relative to Arab patients was 2.33 (95% CI 1.17 to 4.62), the absolute risk difference was 6.3 percentage points (95% CI 1.7 to 10.9 percentage points), and the crude odds ratio was 2.49 (95% CI 1.20 to 5.19). Pearson’s chi-square test of the two-by-two table gave χ^2^ = 6.28, df = 1, *p* = 0.012.

Discharge destination differed between the groups overall (χ^2^ = 7.59, df = 2, *p* = 0.022), but this was accounted for by the mortality difference: among the 457 survivors, the proportions discharged home rather than to institutional care were 48.6% (125/257) in Jewish and 54.0% (108/200) in Arab patients (Pearson’s chi-square test, *p* = 0.255), and median length of stay was 9.0 days (IQR 5.0–14.0) and 8.0 days (IQR 5.0–15.0) respectively (Mann-Whitney *U* test, *p* = 0.567).

### 3.4. Multivariable Models

Table 3 shows the unadjusted association of every recorded admission variable with in-hospital death, together with the multivariable models. In unadjusted analyses, older age, Jewish population group and higher admission NIHSS were associated with higher odds of death and female sex with lower odds, whereas the vascular risk factors, infarct territory, hemispheric side, laboratory values and reperfusion treatments were not. In the primary model containing age, sex, population group and admission NIHSS (Section 2.5), the adjusted odds ratio for Jewish relative to Arab patients was 2.05 (95% CI 0.97 to 4.34; *p* = 0.062). Older age (OR 1.048 per year, 95% CI 1.015 to 1.083; *p* = 0.004) and higher admission NIHSS (OR 1.080 per point, 95% CI 1.009 to 1.155; *p* = 0.026) were each independently associated with higher odds of death, and female sex with lower odds (OR 0.37, 95% CI 0.18 to 0.75; *p* = 0.006). The Hosmer-Lemeshow test gave no indication of gross lack of fit (*p* = 0.859), and discrimination was moderate (C-statistic 0.733, 95% CI 0.642 to 0.813; bootstrap optimism-corrected 0.709).

The statistical significance of the association of population group with mortality was sensitive to model specification. In the extended model, which additionally contained diabetes mellitus, current smoking and log_10_ CRP, the adjusted odds ratio was 2.16 (95% CI 0.99 to 4.70; *p* = 0.053). Substituting log_2_(CRP + 1), which retains the seven patients with a recorded CRP of 0 mg/L and therefore restores the full set of 494 patients with CRP data, gave 2.27 (95% CI 1.04 to 4.95; *p* = 0.039), and the eleven-predictor model gave 2.25 (95% CI 1.02 to 4.94; *p* = 0.044). In that model older age (OR 1.041 per year; *p* = 0.025) and female sex (OR 0.33; *p* = 0.004) remained associated with death, and admission NIHSS was no longer significant (OR 1.063 per point; *p* = 0.096). None of the added vascular risk factors, log_10_ CRP or reperfusion treatments was associated with death (all *p* ≥ 0.137). The point estimate for population group was therefore stable at roughly a doubling of the odds of death, while its position relative to the conventional significance threshold moved with the choice of covariates and of CRP transformation.

### 3.5. Interaction Between Population Group and Prognostic Variables

**Figure 1:**
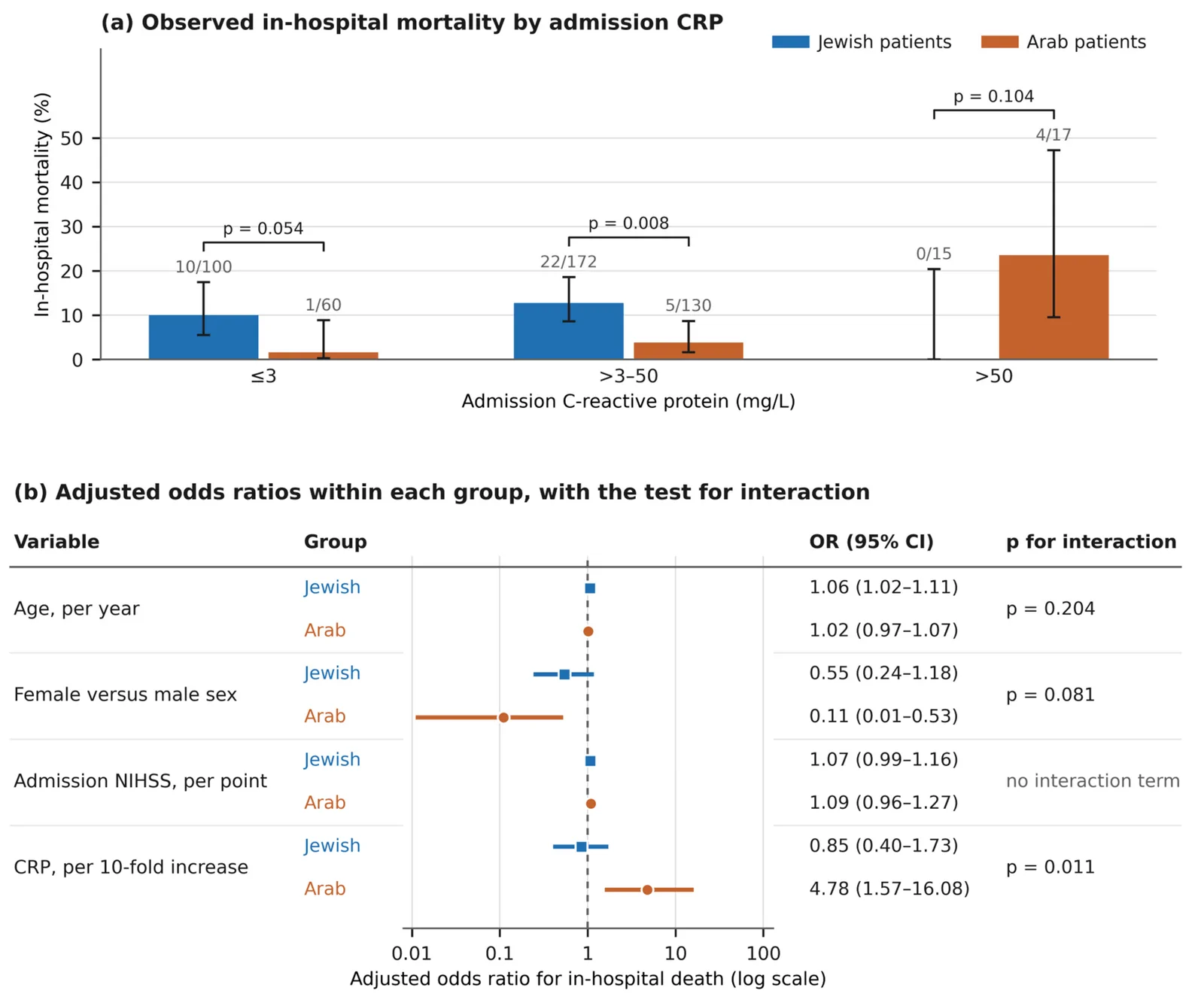
Observed in-hospital mortality by admission C-reactive protein stratum, and adjusted associations of admission variables with in-hospital death, in each population group. (**a**) Observed in-hospital mortality, the percentage of patients who died, within three strata of admission CRP defined on the original scale in mg/L (at or below 3, above 3 up to 50, and above 50 mg/L), among the 494 patients with a recorded CRP (287 Jewish, 207 Arab); the seven patients with CRP of 0 mg/L fall in the lowest stratum. Bars show the observed proportions, error bars their 95% Wilson confidence intervals, and the numbers above each bar the deaths over the patients in that stratum. No death occurred among the 15 Jewish patients with CRP above 50 mg/L. The *p* value above each pair of bars is from Fisher’s exact test comparing the two groups within that stratum; these comparisons are descriptive and unadjusted, and the formal comparison is the interaction estimate in Table 4. (**b**) Adjusted odds ratios for in-hospital death from Firth penalized logistic regression fitted separately within each population group, each model containing age, sex, admission NIHSS and log_10_ CRP (Table 5). Squares mark Jewish patients (*n* = 283; 31 deaths) and circles Arab patients (*n* = 204; 10 deaths); horizontal lines are 95% penalized profile-likelihood confidence intervals. Odds ratios are expressed per additional year of age, for female relative to male sex, per additional NIHSS point and per 10-fold increase in admission CRP, which is one unit on the log_10_ scale (for example, from 5 to 50 mg/L). An odds ratio above 1 indicates higher, and one below 1 lower, odds of death. The horizontal axis is logarithmic, and the dashed vertical reference line marks an odds ratio of 1, that is, no association. The *p* for interaction column gives the penalized likelihood-ratio *p* value for the corresponding population group by variable product term in the pooled model (Table 4); admission NIHSS was not one of the three interaction terms defined for this analysis; its interaction was tested in the exploratory screen (Table S1). Within-group *p* values are given in Table 5 but are not shown here, because the formal comparison between the groups is the interaction test rather than the significance of each group-specific estimate. CI, confidence interval; CRP, C-reactive protein; NIHSS, National Institutes of Health Stroke Scale; OR, odds ratio.

Because only 41 deaths were available and the sex product term approaches separation, penalized estimates are reported as the primary result, with maximum-likelihood estimates alongside them. The joint test of the three interaction terms was significant (penalized likelihood-ratio χ^2^ = 10.96, df = 3, *p* = 0.012; maximum-likelihood χ^2^ = 11.81, *p* = 0.008). Individual interaction estimates are given in Table 4, and the interaction *p* values are shown beside the group-specific estimates in Figure 1. Only the interaction between population group and log_10_ CRP was individually significant (penalized ratio of odds ratios 0.181 per 10-fold increase in CRP, 95% CI 0.046 to 0.671; *p* = 0.011; maximum likelihood 0.164, 95% CI 0.041 to 0.656; *p* = 0.011): the odds ratio for death per 10-fold increase in CRP among Jewish patients was about one fifth of that among Arab patients, so that the association between admission CRP and death was weaker in Jewish patients. The interaction with sex did not reach significance (penalized ratio of odds ratios for female sex 4.893, 95% CI 0.834 to 53.393; *p* = 0.081; maximum likelihood 7.269, 95% CI 0.727 to 72.710; *p* = 0.091). Its direction also pointed toward a weaker association in Jewish patients, the lower odds of death among women being less marked in Jewish than in Arab patients. The interaction with age was not significant (penalized 1.043, 95% CI 0.977 to 1.112; *p* = 0.204; maximum likelihood 1.043, 95% CI 0.976 to 1.115; *p* = 0.212).

In the exploratory screen of the remaining admission variables (Table S1), only white blood cell count showed evidence of an interaction with population group (penalized ratio of odds ratios 0.767 per 1 × 10^9^/L, 95% CI 0.639 to 0.913; *p* = 0.003; false-discovery rate 0.037). Like CRP, it was more weakly associated with death in Jewish than in Arab patients (Table 5). In post hoc checks, this interaction persisted after adjustment for log_10_ CRP (ratio of odds ratios 0.764; *p* = 0.003), with which white blood cell count was only weakly correlated (*r* = 0.10). It also persisted on the log_2_ scale (*p* = 0.018; the false-discovery rate above refers to the untransformed count) and after winsorization at the 95th percentile (*p* = 0.008), and it was attenuated when the five patients with a count above 20 × 10^9^/L, two of them Arab patients who died, were excluded (*p* = 0.050). When the product term for white blood cell count was added to the model containing age, sex, admission NIHSS, log_10_ CRP, white blood cell count, population group and the CRP product term, the white blood cell count interaction remained (*p* = 0.006) and the CRP interaction was attenuated, from a ratio of odds ratios of 0.233 (95% CI 0.063 to 0.828; *p* = 0.024) in that model to 0.290 (95% CI 0.078 to 1.039; *p* = 0.057). The two interactions therefore partly overlap: two of the four Arab patients who died with CRP above 50 mg/L also had counts above 20 × 10^9^/L. None of the other 12 variables, including admission NIHSS, the four vascular risk factors, hemispheric side and both reperfusion treatments, showed evidence of an interaction (all *p* ≥ 0.148).

### 3.6. Group-Stratified Models

Group-stratified penalized models are shown in Table 5 and in Figure 1. Among Jewish patients (283 patients with complete data, 31 deaths), the adjusted odds ratio was 1.062 per year of age (95% CI 1.020 to 1.111; *p* = 0.003), with no significant association for log_10_ CRP (0.85 per 10-fold increase, 95% CI 0.40 to 1.73; *p* = 0.655) or female sex (0.55, 95% CI 0.24 to 1.18; *p* = 0.125). Among Arab patients (204 patients, 10 deaths), the adjusted odds ratios were 4.78 (95% CI 1.57 to 16.08; *p* = 0.006) per 10-fold increase in CRP and 0.11 (95% CI 0.01 to 0.53; *p* = 0.004) for female sex, with no significant association for age (1.018, 95% CI 0.972 to 1.071; *p* = 0.466). Of the variables not included in the adjusted models, two were associated with death among Arab patients in unadjusted analyses: higher white blood cell count (OR 1.190 per 1 × 10^9^/L, 95% CI 1.054 to 1.355; *p* = 0.007) and higher INR (OR 1.361 per 0.1, 95% CI 1.034 to 1.832; *p* = 0.031). None was associated with death among Jewish patients.

Penalization materially changed the magnitude of the estimates in the smaller group: the unpenalized four-predictor odds ratio for female sex among Arab patients was 0.07 (95% CI 0.01 to 0.62) against a penalized 0.11. The penalized estimate is therefore the one reported, and it rests on a contrast of 1 death among 96 Arab women against 9 deaths among 114 Arab men (1.0% versus 7.9%; Fisher’s exact test *p* = 0.023). Among Jewish patients the same contrast was 8.8% versus 13.2% (Fisher’s exact test *p* = 0.264).

**Table 5:** Population-group-stratified Firth penalized logistic regression models for in-hospital death, unadjusted and adjusted.

Variable	Jewish Patients, Unadjusted OR (95% CI); *p*	Jewish Patients, Adjusted OR (95% CI); *p*	Arab Patients, Unadjusted OR (95% CI); *p*	Arab Patients, Adjusted OR (95% CI); *p*
Age, per year	1.062 (1.022–1.109); 0.001	1.062 (1.020–1.111); 0.003	1.013 (0.968–1.066); 0.591	1.018 (0.972–1.071); 0.466
Female sex	0.64 (0.30–1.34); 0.241	0.55 (0.24–1.18); 0.125	0.17 (0.02–0.78); 0.020	0.11 (0.01–0.53); 0.004
Hypertension	1.44 (0.62–3.86); 0.411	—	1.27 (0.34–6.89); 0.738	—
Diabetes mellitus	1.76 (0.84–3.65); 0.134	—	1.32 (0.38–4.60); 0.651	—
Hyperlipidemia	0.85 (0.41–1.82); 0.677	—	0.54 (0.15–1.88); 0.324	—
Current smoking	0.70 (0.22–1.82); 0.487	—	0.86 (0.20–3.01); 0.824	—
NIHSS at admission, per point	1.100 (1.023–1.192); 0.010	1.068 (0.991–1.158); 0.087	1.051 (0.933–1.198); 0.420	1.092 (0.959–1.268); 0.190
Anterior circulation	0.55 (0.22–1.50); 0.227	—	1.05 (0.23–10.02); 0.960	—
Right hemispheric side	0.93 (0.41–2.08); 0.868	—	1.22 (0.34–4.69); 0.758	—
Bilateral or undetermined side	1.68 (0.58–4.41); 0.318	—	1.01 (0.10–5.77); 0.990	—
log_10_ CRP, per 10-fold increase	0.84 (0.41–1.71); 0.638	0.85 (0.40–1.73); 0.655	3.71 (1.32–10.59); 0.013	4.78 (1.57–16.08); 0.006
WBC, per 1 × 10^9^/L	0.927 (0.812–1.047); 0.226	—	1.190 (1.054–1.355); 0.007	—
INR, per 0.1	1.011 (0.761–1.285); 0.932	—	1.361 (1.034–1.832); 0.031	—
PT, per s	1.009 (0.881–1.111); 0.878	—	1.027 (0.978–1.062); 0.197	—
aPTT, per s	0.997 (0.931–1.047); 0.926	—	1.015 (0.924–1.065); 0.653	—
Intravenous thrombolysis	0.96 (0.42–2.04); 0.914	—	0.49 (0.09–1.83); 0.304	—
Mechanical thrombectomy	0.49 (0.17–1.19); 0.119	—	0.69 (0.13–2.61); 0.610	—
Patients (deaths), adjusted model	—	283 (31)	—	204 (10)
C-statistic (95% CI), adjusted model	—	0.716 (0.615–0.813)	—	0.858 (0.755–0.944)

aPTT, activated partial thromboplastin time; CI, confidence interval; CRP, C-reactive protein; INR, international normalized ratio; NIHSS, National Institutes of Health Stroke Scale; OR, odds ratio; PT, prothrombin time; WBC, white blood cell count. Unadjusted odds ratios come from penalized models containing one variable at a time; adjusted odds ratios come from a single penalized model per group containing age, sex, admission NIHSS and log_10_ CRP, fitted to the patients with CRP above 0 mg/L. Values are odds ratios with 95% penalized profile-likelihood confidence intervals, followed by the penalized likelihood-ratio *p* value. Male sex is the reference category for female sex, and left side for hemispheric side. log_10_ CRP is scaled per 10-fold increase in CRP, white blood cell count per 1 × 10^9^/L, INR per 0.1, and prothrombin and activated partial thromboplastin times per second. Prothrombin time and the INR, which is derived from it, are reported only as unadjusted estimates, each from its own model; neither was entered into the adjusted models. Denominators of the unadjusted estimates vary slightly with missing values. The C-statistic row refers to the adjusted models and reports bootstrap percentile confidence intervals. These stratified models are presented to aid interpretation of the interaction estimates in Table 4 and should not be read as independent evidence that an association is present in one group and absent in the other. Exact *p* values are given to three decimal places; *p* values below 0.001 are shown as <0.001.

### 3.7. Calibration of a Pooled Model Within Each Group

A pooled model containing the same four admission variables as the interaction and stratified models (age, sex, admission NIHSS and log_10_ CRP; Section 2.5), without any population-group term, was fitted to the 487 patients with CRP above 0 mg/L and achieved a C-statistic of 0.721 (95% CI 0.632 to 0.800). Within this model the odds ratio per 10-fold increase in CRP was 1.29 (95% CI 0.70 to 2.35; *p* = 0.416).

**Figure 2:**
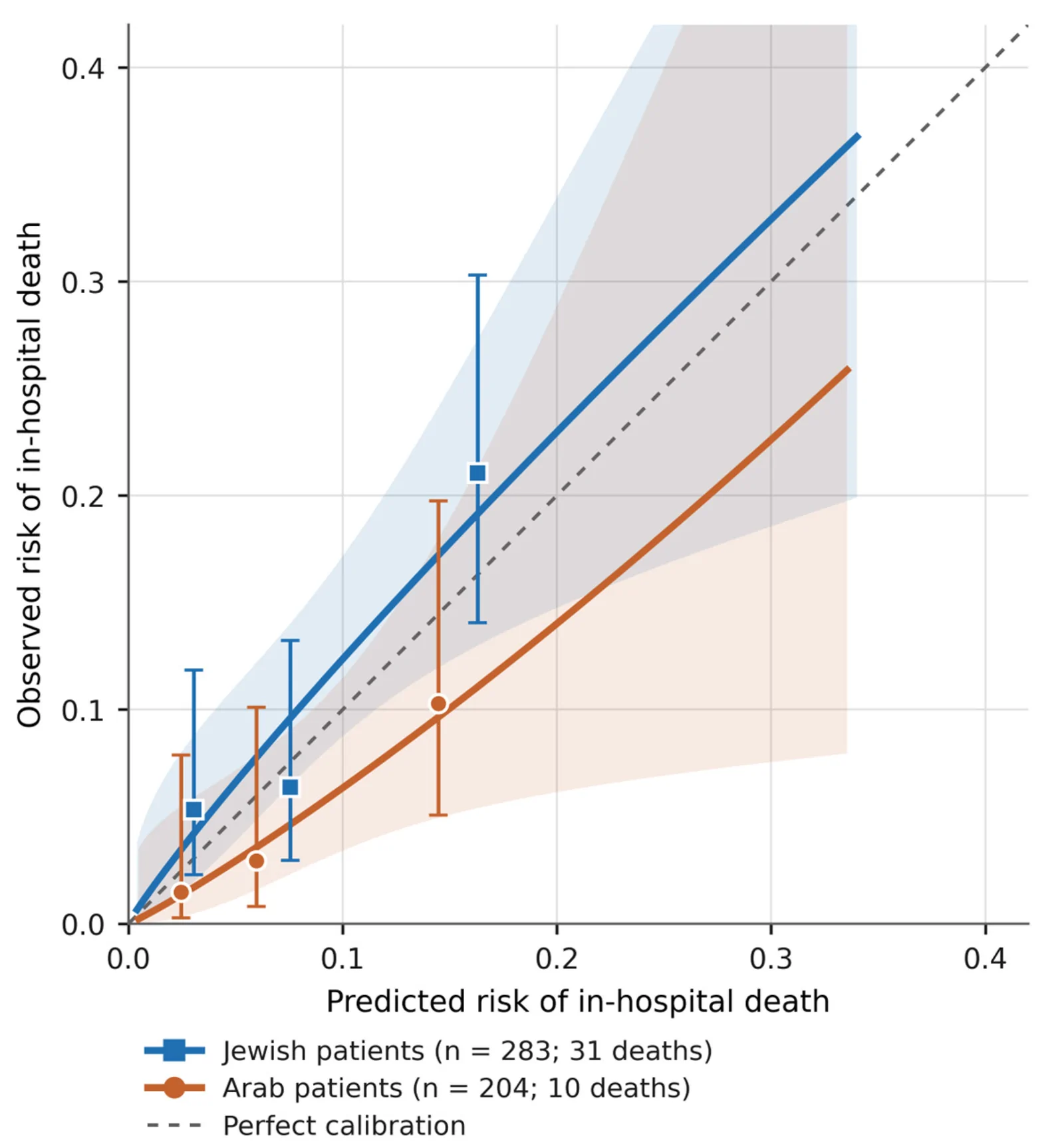
Calibration of a prognostic model fitted without a population-group term, applied within each population group. The model contains age, sex, admission NIHSS and log_10_ CRP (per 10-fold increase in CRP) and no population-group term, and was fitted to the 487 patients with CRP above 0 mg/L. The horizontal axis is the probability of in-hospital death predicted by that model and the vertical axis the observed probability, both on a linear scale from 0 to 0.4. The dashed diagonal is the line of identity, on which predicted and observed risk agree (perfect calibration). The solid lines are logistic recalibration curves, obtained within each group by regressing the observed outcome on the model’s linear predictor, and the shaded bands are their 95% pointwise confidence intervals. The markers are the observed proportions of death within tertiles of predicted risk, plotted at the mean predicted risk of each tertile, with 95% Wilson confidence intervals. A curve above the diagonal indicates underprediction and a curve below it overprediction. The model predicted 25.4 deaths among Jewish patients, where 31 occurred (observed-to-expected ratio 1.22, 95% CI 0.86 to 1.73), and 15.6 among Arab patients, where 10 occurred (ratio 0.64, 95% CI 0.35 to 1.19). There was no evidence of a difference in calibration slope between the groups (*p* = 0.751), while under a common slope the calibration level did differ (*p* = 0.048): within this cohort the model underpredicted deaths in Jewish patients and overpredicted them in Arab patients. Because the model was fitted and evaluated in the same cohort, this is an internal, exploratory comparison of subgroup calibration rather than an external validation. CI, confidence interval; CRP, C-reactive protein; NIHSS, National Institutes of Health Stroke Scale.

Applied within each group, the pooled model discriminated to a similar degree in both, with C-statistics of 0.702 (95% CI 0.592 to 0.803) in Jewish and 0.753 (95% CI 0.588 to 0.887) in Arab patients, but its absolute risk estimates were displaced in opposite directions (Figure 2): it predicted 25.4 deaths among Jewish patients where 31 occurred (observed-to-expected ratio 1.22, 95% CI 0.86 to 1.73) and 15.6 deaths among Arab patients where 10 occurred (ratio 0.64, 95% CI 0.35 to 1.19). Each interval taken alone includes unity; the formal comparison between the groups is given below. The two components of calibration behaved differently. There was no evidence of a difference in calibration slope between the groups (0.92, 95% CI 0.41 to 1.44, in Jewish and 1.08, 95% CI 0.24 to 1.93, in Arab patients; likelihood-ratio test of difference *p* = 0.751), although both estimates are imprecise, so this is an absence of a detectable difference rather than a demonstration of equality. The calibration level did differ: under a common slope, adding a population-group term to the recalibration model gave 0.73 on the log-odds scale (95% profile-likelihood interval 0.01 to 1.53; likelihood-ratio χ^2^ = 3.90, df = 1, *p* = 0.048), equivalent to a 2.07-fold difference in the odds of death at the same predicted risk. Within this cohort the pooled model is therefore displaced in level: it underpredicted deaths in Jewish patients and overpredicted them in Arab patients, with no detectable difference in the steepness of the relationship between predicted and observed risk.

### 3.8. Sensitivity Analyses

Sensitivity analyses are summarized in Table 6. The interaction between population group and CRP was robust to winsorization at the 95th percentile (penalized ratio of odds ratios 0.154; *p* = 0.012), to the log_2_(CRP + 1) transformation, which retains the seven patients with CRP of 0 mg/L and is scaled per doubling of (CRP + 1) rather than per 10-fold increase in CRP (0.541 per doubling; *p* = 0.006), to restriction of CRP to values at or below 100 mg/L (0.199; *p* = 0.039), to exclusion of thrombolysis recipients (0.128; *p* = 0.010), to exclusion of thrombectomy recipients (0.121; *p* = 0.009), to restriction to patients aged 50 years or older (0.174; *p* = 0.009), and to multiple imputation (maximum-likelihood ratio of odds ratios 0.165, 95% CI 0.041 to 0.662; *p* = 0.011).

It was, however, strongly attenuated when analysis was restricted to CRP values at or below 50 mg/L (0.538, 95% CI 0.068 to 4.103; *p* = 0.547) and was not detected when CRP was dichotomized at the conventional 3 mg/L threshold (0.438; *p* = 0.380). The reason is visible in the raw counts, shown in Figure 1a. At or below 50 mg/L, mortality was higher in Jewish than in Arab patients, significantly so in the stratum above 3 up to 50 mg/L (12.8% versus 3.8%; Fisher’s exact test *p* = 0.008) and at the margin of significance in the stratum at or below 3 mg/L (10.0% versus 1.7%; *p* = 0.054). Above 50 mg/L the direction reversed: none of the 15 Jewish patients died, whereas 4 of the 17 Arab patients did (23.5%; *p* = 0.104). These stratum-specific comparisons are descriptive and unadjusted, but the reversal is what the interaction estimate detects. The heterogeneity we detected is therefore concentrated in the upper tail of the inflammatory distribution.

For the population-group difference itself, propensity-score matching on age, sex, hypertension, diabetes mellitus, hyperlipidemia, current smoking, admission NIHSS and log_10_ CRP matched 185 of the 203 Arab patients with complete data on the matching variables to a Jewish patient, so that the matched comparison describes patients with the covariate profile of the Arab group. All standardized mean differences fell below 0.12 after matching, from a maximum of 0.41 before matching (Table S2). Mortality was 9.7% (18/185) among matched Jewish and 4.9% (9/185) among matched Arab patients. In 17 pairs only the Jewish patient died and in 8 only the Arab patient. The resulting conditional odds ratio was 2.13 (95% CI 0.87 to 5.69; exact McNemar *p* = 0.108), in the same direction as, and of similar magnitude to, the adjusted estimates, but with a confidence interval that includes unity. Among the 225 patients who received neither thrombolysis nor thrombectomy, mortality was 13.2% (18/136) in Jewish and 6.7% (6/89) in Arab patients (Pearson’s chi-square test, *p* = 0.123). Under multiple imputation the extended model gave an adjusted odds ratio for Jewish population group of 2.28 (95% CI 1.05 to 4.96; *p* = 0.037), against 2.16 in the complete-case analysis.

## 4. Discussion

### 4.1. Principal Findings

In a single-center cohort of 499 patients with non-lacunar acute ischemic stroke and moderate or greater vessel occlusion, in-hospital mortality among Jewish patients was more than twice that among Arab patients, in the context of an older age at onset and despite a lighter smoking burden and a non-significantly lower prevalence of diabetes mellitus. The crude difference was statistically significant (risk ratio 2.33, 95% CI 1.17 to 4.62). The adjusted odds ratio was 2.05 in the primary model (95% CI 0.97 to 4.34) and stayed between 2.05 and 2.28 when the four vascular risk factors, CRP and the two reperfusion treatments were added, when CRP was transformed differently and under multiple imputation (Table 3 and Table 6), and the direction persisted in propensity-matched pairs. The second finding concerns prognosis: in this cohort, the same admission variables did not carry the same prognostic weight in the two groups, which bears directly on whether a risk estimate produced for one population can be applied to another.

Our crude difference is larger than national data would lead one to expect. In national series, Arab patients present younger [19] and with more diabetes mellitus and smoking [17,18], a pattern our cohort shared, and the registry analysis found comparable interventional treatment, outcome and mortality between the groups [18]. That analysis was, however, restricted to revascularized patients, a selected minority of all patients with stroke, and therefore answers a different question. The comparison argues for the registry-wide replication proposed below rather than for treating 11.1% versus 4.8% as a regional rate.

In-hospital death after stroke reflects not only infarct severity but also decisions to limit treatment, which vary between settings and populations for reasons that routine records do not capture. Our data are compatible with such a mechanism, since admission severity and reperfusion rates were similar and the mortality difference persisted in direction after adjustment and after propensity matching. Treatment-limitation and do-not-resuscitate decisions were not recorded in this dataset, so we can neither support nor exclude this explanation.

### 4.2. Heterogeneity in the Prognostic Value of Admission C-Reactive Protein

Of the three interaction terms defined for this analysis, the main signal was for admission CRP, the only one to reach significance on its own: its association with in-hospital death was weaker in Jewish than in Arab patients, on a formal interaction test rather than by inference from group-specific significance. CRP after acute ischemic stroke is reproducibly associated with poor outcome and death [8,9], but in a large prospective cohort, adding inflammatory markers to a clinical prediction model did not materially improve discrimination or reclassification [12]. Our data suggest one possible reason: if a marker carries prognostic information in some populations and little in others, its average incremental value across a mixed cohort will be small even where it is substantial in a subgroup. The pooled model in this cohort behaved in that way, assigning CRP a weight close to the null.

This interaction should be interpreted with care. It was concentrated in the upper tail of the CRP distribution: none of the 15 Jewish patients with CRP above 50 mg/L died, against 4 of the 17 Arab patients, and the interaction was strongly attenuated when CRP was restricted to 50 mg/L or less and was not detected when CRP was dichotomized at 3 mg/L. It was also attenuated when the interaction of population group with white blood cell count was modeled alongside it (Section 3.5). The finding therefore concerns patients presenting with a marked systemic inflammatory response. After stroke, such a response may reflect infarct size, concurrent infection, pre-existing inflammatory or metabolic conditions or autonomic regulation of inflammation; in an earlier cohort from this service, vagally mediated cardiac activity on admission was related to short-term clinical outcome after stroke and differed by the side of the lesion [44]. Baseline CRP distributions also differ between population groups [13,14,15], but a difference in distribution is not a difference in prognostic meaning, and the present finding does not justify interpreting a given CRP value differently according to population group in clinical practice.

### 4.3. Sex and Age

The interactions with sex and age were not individually significant (Table 4). Among Arab patients, the association of female sex with lower mortality rests on a single death among 96 women, and sex differences in stroke outcome are shaped by age at onset, comorbidity and care pathways as much as by biology [45,46]. For age, the coefficient reached significance in the Jewish stratum and not in the Arab one, which is a difference in significance and not a difference in association [33]. The Arab age distribution was shifted downward (43.8% of Arab and 59.9% of Jewish patients were older than 70 years), placing much of the Arab group in a range where few deaths occurred. A difference in case mix, rather than a different biology of aging, is the most parsimonious explanation.

### 4.4. Other Admission Variables

In unadjusted analyses of the whole cohort, only age, sex, population group and admission NIHSS were associated with in-hospital death (Table 3), and none of the vascular risk factors, CRP or reperfusion treatments added in the eleven-predictor model was associated with death after adjustment. Smoking and, less clearly, diabetes mellitus were more common among Arab patients, but neither was associated with death, and accounting for them did not reduce the population-group estimate. The exploratory screen identified one further variable whose prognostic association differed between the groups: white blood cell count, the other inflammatory marker measured at admission, which was associated with death among Arab patients (unadjusted OR 1.190 per 1 × 10^9^/L) but not detectably among Jewish patients (0.927). Modeled together with CRP, its interaction remained while that of CRP was attenuated, so the two signals partly overlap and rest partly on the same deaths among Arab patients. The heterogeneity may therefore concern the systemic inflammatory response rather than CRP alone, a possibility that requires confirmation. No other variable, including stroke severity, the vascular risk factors, hemispheric side and reperfusion treatment, showed evidence of a different prognostic association (Table S1), although such tests have limited power.

### 4.5. Implications for Personalized Medicine

The implication for personalized medicine is specific. It is not that Jewish and Arab patients should be treated differently. Population group here is an administrative and social category, not a biological one [22,23], and none of our analyses identifies a mechanism that would justify group-based therapeutic decisions. Acute stroke treatment should continue to follow guideline criteria applied identically to all patients [47].

What the data do suggest is that the machinery of individualized risk estimation deserves local scrutiny. A pooled model built on this cohort discriminated similarly in both groups, but in this internal comparison its calibration level differed between them. If confirmed externally, that combination would point to recalibration rather than redevelopment [48,49]. In a service that used such a model to trigger escalation of monitoring or to frame a goals-of-care conversation, a displacement of this kind would be felt by individual patients. The remedy is not a separate model per group but the standard apparatus of prediction-model research applied with population composition in view: reporting calibration as well as discrimination [32], testing case-mix relatedness before assuming transportability [1,48], and recalibrating locally where warranted [32,49]. By the same logic, a CRP threshold validated in one population should be re-examined before it is adopted in another.

### 4.6. Strengths and Limitations

The main strength of this study is its setting. Both groups were admitted to one regional stroke center during one period and treated by the same clinicians under one set of protocols, so the differences between hospitals, staffing and infrastructure that confound most comparisons of this kind are largely removed by design. Admission stroke severity and rates of thrombolysis and thrombectomy were comparable, with no shortfall in thrombolysis among Arab patients of the kind reported nationally [18], and similar length of stay and discharge disposition among survivors make differential discharge or transfer practice an unlikely explanation. Prognostic heterogeneity was assessed by explicit interaction tests, every recorded admission variable was examined, and sparse data were handled by penalized likelihood.

Ten deaths among Arab patients is a small number of events. It supports a descriptive mortality rate and a direction of association, but not precise subgroup estimation, and interaction tests are markedly less powerful than tests of main effects [33,50], particularly in the exploratory screen. The study is retrospective and single-center, so residual confounding cannot be excluded. The cohort is a defined clinical subgroup rather than a consecutive series: lacunar infarcts and strokes with only mild vessel occlusion were excluded when the source database was compiled, and the cohort is weighted toward moderate-to-severe stroke (median admission NIHSS 14; 55% received reperfusion treatment). It therefore represents a population in which in-hospital mortality risk is routinely estimated at a thrombectomy-capable center, and the findings apply to non-lacunar stroke with moderate or greater vessel occlusion rather than to all ischemic strokes in northern Israel. We had no data on time from symptom onset to arrival, language of consultation or socioeconomic position [51,52], any of which could contribute to a difference in in-hospital death. Stroke etiology by the Trial of Org 10172 in Acute Stroke Treatment (TOAST) classification, the site and degree of vessel occlusion, reperfusion success, infarct volume, pre-stroke functional status and in-hospital infection were also not available in the analysis dataset, and the timing of the CRP sample relative to reperfusion was not recorded. Arab subgroups could not be distinguished, although clinically meaningful heterogeneity between them has been reported in this region [21]. The CRP interaction rested on few patients and four deaths in the upper tail of the distribution, so its magnitude should be regarded as unstable until replicated. Follow-up ended at discharge, so early post-discharge deaths were not captured.

### 4.7. Future Directions

Three steps follow. The mortality comparison should be repeated across the Israeli stroke registry, where the number of events would allow adjusted estimation with useful precision. Existing stroke prognostic models should then be evaluated for calibration separately within Jewish and Arab patients in a multicenter dataset, reporting observed-to-expected ratios and calibration slopes alongside discrimination. Finally, the inflammatory hypothesis should be tested prospectively with infarct volume, stroke etiology, systematic surveillance for infection and serial CRP and white blood cell measurements timed relative to reperfusion, in a cohort large enough to estimate the interaction rather than merely detect it.

## 5. Conclusions

Among 499 patients with non-lacunar acute ischemic stroke and moderate or greater vessel occlusion treated at one regional center in northern Israel, in-hospital mortality was more than twice as high in Jewish as in Arab patients. The difference was statistically significant in crude terms, and after adjustment for case mix the point estimate remained close to a doubling of the odds of death, although its confidence interval included unity in the primary model. A second observation bears more directly on personalized medicine: the same admission variables carried different prognostic information in the two groups, mainly because admission CRP was more weakly associated with death in Jewish than in Arab patients. An exploratory analysis found a similar, partly overlapping pattern for white blood cell count, and the interactions with age and sex were not individually significant.

These findings come from a single-center observational study with few deaths among Arab patients; they are exploratory and hypothesis-generating, and they cannot establish why the two groups differ. Their value for personalized medicine lies in the question they raise: whether a risk estimate generated by a pooled model is equally trustworthy for every patient to whom it is applied. In this cohort, whose two population groups shared one hospital, one insurance system and one set of protocols, the data suggest that it was not. What follows is a research agenda, not a change in practice: external replication, followed by validation and local recalibration of stroke prognostic models, with treatment decisions left to guideline criteria applied identically to all patients.

## Figures and Tables

**Table 3:** Unadjusted and multivariable logistic regression models for in-hospital death.

Variable	Unadjusted OR (95% CI); *p*	Model 1 (Primary), OR (95% CI); *p* ^a^	Model 2 (Extended), OR (95% CI); *p* ^b^	Model 3 (Eleven Predictors), OR (95% CI); *p* ^c^
Age, per year	1.049 (1.018–1.081); 0.002	1.048 (1.015–1.083); 0.004	1.045 (1.009–1.082); 0.013	1.041 (1.005–1.079); 0.025
Female sex	0.48 (0.24–0.95); 0.036	0.37 (0.18–0.75); 0.006	0.37 (0.18–0.75); 0.006	0.33 (0.16–0.70); 0.004
Jewish population group	2.49 (1.20–5.19); 0.015	2.05 (0.97–4.34); 0.062	2.16 (0.99–4.70); 0.053	2.25 (1.02–4.94); 0.044
Hypertension	1.54 (0.69–3.41); 0.292	—	—	1.34 (0.52–3.44); 0.546
Diabetes mellitus	1.51 (0.80–2.84); 0.206	—	1.62 (0.83–3.18); 0.161	1.73 (0.84–3.55); 0.137
Hyperlipidemia	0.76 (0.40–1.43); 0.392	—	—	0.59 (0.28–1.23); 0.157
Current smoking	0.58 (0.25–1.34); 0.204	—	0.82 (0.29–2.27); 0.701	0.95 (0.34–2.67); 0.917
NIHSS at admission, per point	1.094 (1.025–1.169); 0.007	1.080 (1.009–1.155); 0.026	1.077 (1.004–1.155); 0.038	1.063 (0.989–1.143); 0.096
Anterior circulation	0.73 (0.31–1.71); 0.466	—	—	—
Right hemispheric side ^d^	0.96 (0.48–1.92); 0.908	—	—	—
Bilateral or undetermined side ^d^	1.32 (0.53–3.30); 0.547	—	—	—
log_10_ CRP, per 10-fold increase	1.20 (0.67–2.16); 0.540	—	1.41 (0.76–2.62); 0.271	1.39 (0.74–2.63); 0.310
WBC, per 1 × 10^9^/L	1.031 (0.943–1.127); 0.506	—	—	—
INR, per 0.1	1.134 (0.936–1.373); 0.200	—	—	—
PT, per s	1.011 (0.961–1.062); 0.679	—	—	—
aPTT, per s	0.991 (0.945–1.038); 0.691	—	—	—
Intravenous thrombolysis	0.75 (0.37–1.50); 0.418	—	—	0.82 (0.39–1.74); 0.612
Mechanical thrombectomy	0.48 (0.21–1.12); 0.089	—	—	0.53 (0.21–1.35); 0.184
Patients (deaths)	—	499 (42)	487 (41)	483 (41)
C-statistic (95% CI)	—	0.733 (0.642–0.813)	0.738 (0.660–0.809)	0.756 (0.689–0.818)
Nagelkerke R^2^	—	0.127	0.134	0.151
Hosmer-Lemeshow *p*	—	0.859	0.603	0.802

aPTT, activated partial thromboplastin time; CI, confidence interval; CRP, C-reactive protein; INR, international normalized ratio; NIHSS, National Institutes of Health Stroke Scale; OR, odds ratio; PT, prothrombin time; WBC, white blood cell count. Arab population group is the reference category for population group, and male sex for female sex. Unadjusted estimates come from univariable logistic regression, so the Wald *p* value for population group differs slightly from the Pearson chi-square *p* value for in-hospital death in Table 1. log_10_ CRP is scaled per 10-fold increase in CRP, white blood cell count per 1 × 10^9^/L, INR per 0.1, and prothrombin and activated partial thromboplastin times per second. Because each unadjusted estimate comes from a model containing one variable alone, prothrombin time and the INR, which is derived from it, are both reported in the unadjusted column; neither was entered into Models 1 to 3. The Hosmer-Lemeshow *p* value is a coarse test of goodness of fit and is reported only to show that there was no indication of gross lack of fit; calibration of a pooled model without a population-group term is assessed in Section 3.7. ^a^ Primary model: age, sex, population group and admission NIHSS; no missing data. Bootstrap optimism-corrected C-statistic 0.709; bootstrap 95% CI for the apparent C-statistic 0.642 to 0.813. ^b^ Extended model additionally containing diabetes mellitus, current smoking and log_10_ CRP; restricted to the 487 patients with CRP above 0 mg/L. Substituting log_2_(CRP + 1), which retains the seven patients with CRP of 0 mg/L, gives an odds ratio for Jewish population group of 2.27 (95% CI 1.04 to 4.95; *p* = 0.039) in 494 patients. ^c^ Eleven-predictor model containing age, sex, population group, hypertension, diabetes mellitus, hyperlipidemia, current smoking, admission NIHSS, log_10_ CRP, intravenous thrombolysis and mechanical thrombectomy, fitted to the 483 patients with complete data (41 deaths); with fewer than four deaths per predictor, its individual coefficients are interpreted with caution. ^d^ Left side is the reference category; 64 of the 65 posterior-circulation strokes were coded as bilateral or undetermined, so hemispheric side was not entered into the multivariable models. Exact *p* values are given to three decimal places; *p* values below 0.001 are shown as <0.001.

**Table 4:** Interaction estimates between population group and candidate prognostic variables.

Interaction Term	Firth Penalized, ROR (95% CI) ^a^	*p* ^a^	Maximum Likelihood, ROR (95% CI) ^b^	*p* ^b^
Jewish group × age, per year	1.043 (0.977–1.112)	0.204	1.043 (0.976–1.115)	0.212
Jewish group × female sex	4.893 (0.834–53.393)	0.081	7.269 (0.727–72.710)	0.091
Jewish group × log_10_ CRP, per 10-fold increase	0.181 (0.046–0.671)	0.011	0.164 (0.041–0.656)	0.011
Joint test of all three terms	χ^2^ = 10.96, df = 3	0.012	χ^2^ = 11.81, df = 3	0.008

CI, confidence interval; CRP, C-reactive protein; df, degrees of freedom; ROR, ratio of odds ratios. Values are ratios of odds ratios with 95% confidence intervals, except the final row, which gives the joint likelihood-ratio test statistic. All estimates come from a single model fitted to the 487 patients with CRP above 0 mg/L (41 deaths), containing age, sex, admission NIHSS, log_10_ CRP, population group and the three product terms. A ratio of odds ratios is the odds ratio among Jewish patients divided by the odds ratio among Arab patients. For age and CRP, a value below 1 means a smaller odds ratio, that is, a weaker positive association with death, among Jewish patients; for female sex, whose odds ratios are below 1 in both groups, a value above 1 means a weaker inverse association among Jewish patients. Penalized estimates are the primary result because the sex product term approaches separation with 41 deaths; maximum-likelihood estimates are given alongside them. ^a^ Penalized profile-likelihood confidence intervals and penalized likelihood-ratio *p* values. ^b^ Wald confidence intervals and Wald *p* values; the joint test is a likelihood-ratio test. Exact *p* values are given to three decimal places; *p* values below 0.001 are shown as <0.001.

**Table 6:** Sensitivity analyses of the interaction between population group and CRP and of the population-group difference in mortality.

Analysis	Patients (Deaths)	Estimate (95% CI Where Applicable)	*p*
**Interaction between population group and CRP**			
Primary specification, log_10_ CRP ^a^	487 (41)	ROR 0.181 (0.046–0.671)	0.011
CRP restricted to ≤100 mg/L	475 (40)	ROR 0.199 (0.038–0.925)	0.039
CRP restricted to ≤50 mg/L	455 (37)	ROR 0.538 (0.068–4.103)	0.547
CRP winsorized at the 95th percentile	487 (41)	ROR 0.154 (0.031–0.673)	0.012
log_2_(CRP + 1), retains CRP = 0	494 (42)	ROR 0.541 (0.341–0.842)	0.006
CRP dichotomized at 3 mg/L	494 (42)	ROR 0.438 (0.041–2.542)	0.380
Excluding intravenous thrombolysis	321 (29)	ROR 0.128 (0.021–0.622)	0.010
Excluding mechanical thrombectomy	346 (34)	ROR 0.121 (0.020–0.597)	0.009
Restricted to age ≥50 years	456 (40)	ROR 0.174 (0.044–0.648)	0.009
Multiple imputation, 20 imputations ^b^	494 (42)	ROR 0.165 (0.041–0.662)	0.011
**Population-group difference in mortality**			
Crude comparison	499 (42)	RR 2.33 (1.17–4.62)	0.012
Primary adjusted model	499 (42)	OR 2.05 (0.97–4.34)	0.062
Propensity-score matched pairs ^c^	370 (27)	9.7% versus 4.9%; conditional OR 2.13 (0.87–5.69)	0.108
Neither thrombolysis nor thrombectomy	225 (24)	13.2% versus 6.7%	0.123
Extended model, multiple imputation ^b^	494 (42)	OR 2.28 (1.05–4.96)	0.037

CI, confidence interval; CRP, C-reactive protein; OR, odds ratio; ROR, ratio of odds ratios; RR, risk ratio. Interaction estimates are Firth penalized ratios of odds ratios (maximum likelihood in the multiple-imputation row) for the population group by CRP product term, each from a model with the same covariates as the primary interaction model in Table 4 (age, sex, admission NIHSS, CRP, population group and the three product terms), so that each row changes only the element named; a value below 1 indicates a weaker CRP-mortality association among Jewish patients. The corresponding maximum-likelihood estimate for the primary specification is 0.164 (95% CI 0.041 to 0.656). All CRP terms are scaled per 10-fold increase in CRP except the log_2_(CRP + 1) row, which is scaled per doubling of (CRP + 1), and the binary row, which contrasts CRP above 3 mg/L with CRP at or below 3 mg/L; the three are therefore not directly comparable in magnitude. The two exclusion rows also drop patients whose reperfusion status was not recorded (two patients for thrombectomy). The two rows reported as percentages give Jewish versus Arab in-hospital mortality. ^a^ Firth penalized estimate; the corresponding maximum-likelihood value is given above. ^b^ Multiple imputation by chained equations with 20 imputations, pooled by Rubin’s rules; estimates are maximum likelihood. ^c^ 185 matched pairs formed by 1:1 nearest-neighbor matching without replacement on age, sex, hypertension, diabetes mellitus, hyperlipidemia, current smoking, admission NIHSS and log_10_ CRP, with a caliper of 0.2 standard deviations of the logit propensity score; all standardized mean differences after matching were below 0.12, compared with a maximum of 0.41 before matching (Table S2). The conditional odds ratio is the ratio of discordant pairs (17 in which only the Jewish patient died and 8 in which only the Arab patient died), and the *p* value is from an exact McNemar test. Exact *p* values are given to three decimal places; *p* values below 0.001 are shown as <0.001.

## Data Availability

The de-identified dataset analyzed in this study is available from the corresponding author upon reasonable request, subject to appropriate data sharing agreements and institutional approvals. Restrictions apply because the data were collected as part of routine clinical care at a single institution.

## Supplementary Materials

The following supporting information can be downloaded at: https://www.mdpi.com/article/10.3390/jpm16100535/s1, Table S1: Exploratory screen of interactions between population group and the remaining admission variables; Table S2: Covariate balance before and after propensity-score matching.
