# Supplementary material for: Same Hospital, Different Outcome: In-Hospital Mortality and Divergent Prognostic Associations in Jewish and Arab Patients with Acute Ischemic Stroke in Northern Israel
Source: J Pers Med. 2026 Oct 20;16(10):535. doi: 10.3390/jpm16100535 (PMC13705706; doi:10.3390/jpm16100535)
Supplement: Supplementary file 1 [file jpm-16-00535-s001.zip › jpm-4549917-supplementary.pdf]

Supplementary Materials

Same Hospital, Different Outcome: In-Hospital Mortality and Divergent Prognostic Associations in Jewish and Arab Patients with Acute Ischemic Stroke in Northern Israel

Table S1. Exploratory screen of interactions between population group and the remaining admission variables.

Variable	Patients (deaths)	Ratio of odds ratios, Jewish versus Arab (95% CI)	p	False-discovery rate
Hypertension	498 (42)	1.294 (0.194–6.922)	0.773	0.914
Diabetes mellitus	499 (42)	1.407 (0.323–6.114)	0.645	0.914
Hyperlipidemia	498 (42)	1.762 (0.405–7.716)	0.445	0.914
Current smoking	499 (42)	1.001 (0.180–5.755)	0.999	0.999
NIHSS at admission, per point	499 (42)	1.031 (0.884–1.192)	0.689	0.914
Anterior circulation	499 (42)	0.491 (0.042–3.325)	0.485	0.914
Hemispheric side (right and bilateral or undetermined versus left)	499 (42)	— (χ^2 = 0.62, df = 2)	0.734	0.914
WBC, per 1 × 10^9/L	499 (42)	0.767 (0.639–0.913)	0.003	0.037
INR, per 0.1	489 (41)	0.756 (0.505–1.107)	0.148	0.914
PT, per s	497 (42)	0.997 (0.847–1.122)	0.967	0.999
aPTT, per s	498 (42)	0.959 (0.879–1.079)	0.412	0.914
Intravenous thrombolysis	499 (42)	1.589 (0.325–10.112)	0.579	0.914
Mechanical thrombectomy	497 (42)	0.733 (0.133–4.963)	0.731	0.914

Each row comes from a separate Firth penalized logistic regression model containing age, sex, admission NIHSS, the listed variable, population group and the product of population group and the listed variable; for admission NIHSS, the product term was added to a model that already contained it. The ratio of odds ratios is the odds ratio for the listed variable among Jewish patients divided by that among Arab patients, so that a value below 1 indicates a smaller odds ratio among Jewish patients. Confidence intervals are penalized profile-likelihood intervals and p values penalized likelihood-ratio tests. Hemispheric side, a three-category variable, was tested with two product terms and a likelihood-ratio test on two degrees of freedom, so no single ratio of odds ratios is given. False-discovery rates are Benjamini-Hochberg adjusted values across the 13 tests. The interactions of population group with age, sex and $\log_{10}$ CRP are reported in Table 4 of the main text. Post hoc checks for white blood cell count: the interaction persisted after adjustment for $\log_{10}$ CRP, 0.764 (95% CI 0.636 to 0.911; p = 0.003); on the $\log_2$ scale, 0.183 per doubling (95% CI 0.044 to 0.748; p = 0.018); and after winsorization at the 95th percentile, 0.734 (95% CI 0.577 to 0.925; p = 0.008). It was attenuated when the five patients with a count above 20 × 10^9/L were excluded, 0.789 (95% CI 0.620 to 1.000; p = 0.050). In a joint model containing age, sex, admission NIHSS, $\log_{10}$ CRP, white blood cell count, population group and both product terms, the ratio of odds ratios for white blood cell count was 0.780 (95% CI 0.649 to 0.931; p = 0.006) and that for $\log_{10}$ CRP was 0.290 (95% CI 0.078 to 1.039; p = 0.057). In the same model without the product term for white blood cell count, the ratio of odds ratios for $\log_{10}$ CRP was 0.233 (95% CI 0.063 to 0.828; p = 0.024). aPTT, activated partial thromboplastin time; INR, international normalized ratio; NIHSS, National Institutes of Health Stroke Scale; PT, prothrombin time; WBC, white blood cell count.

Table S2. Covariate balance before and after propensity-score matching.

Variable	Standardized mean difference before matching	Standardized mean difference after matching
Age	+0.340	+0.071
Female sex	+0.024	+0.076
Hypertension	+0.005	+0.025
Diabetes mellitus	−0.169	+0.043

Variable	Standardized mean difference before matching	Standardized mean difference after matching
Hyperlipidemia	−0.044	+0.056
Current smoking	−0.409	−0.119
NIHSS at admission	+0.071	+0.027
$\log_{10}$ CRP	−0.265	+0.008

Standardized mean differences are the difference between Jewish and Arab patients divided by the pooled standard deviation, computed among the 485 patients with complete matching variables (282 Jewish, 203 Arab) before matching and among the 185 matched pairs after matching. Each Arab patient was matched without replacement to the Jewish patient with the closest logit propensity score within a caliper of 0.2 standard deviations; 18 Arab patients had no match within the caliper. A positive value indicates a higher mean or proportion among Jewish patients. $\log_{10}$ CRP is scaled per 10-fold increase in CRP. CRP, C-reactive protein; NIHSS, National Institutes of Health Stroke Scale.
